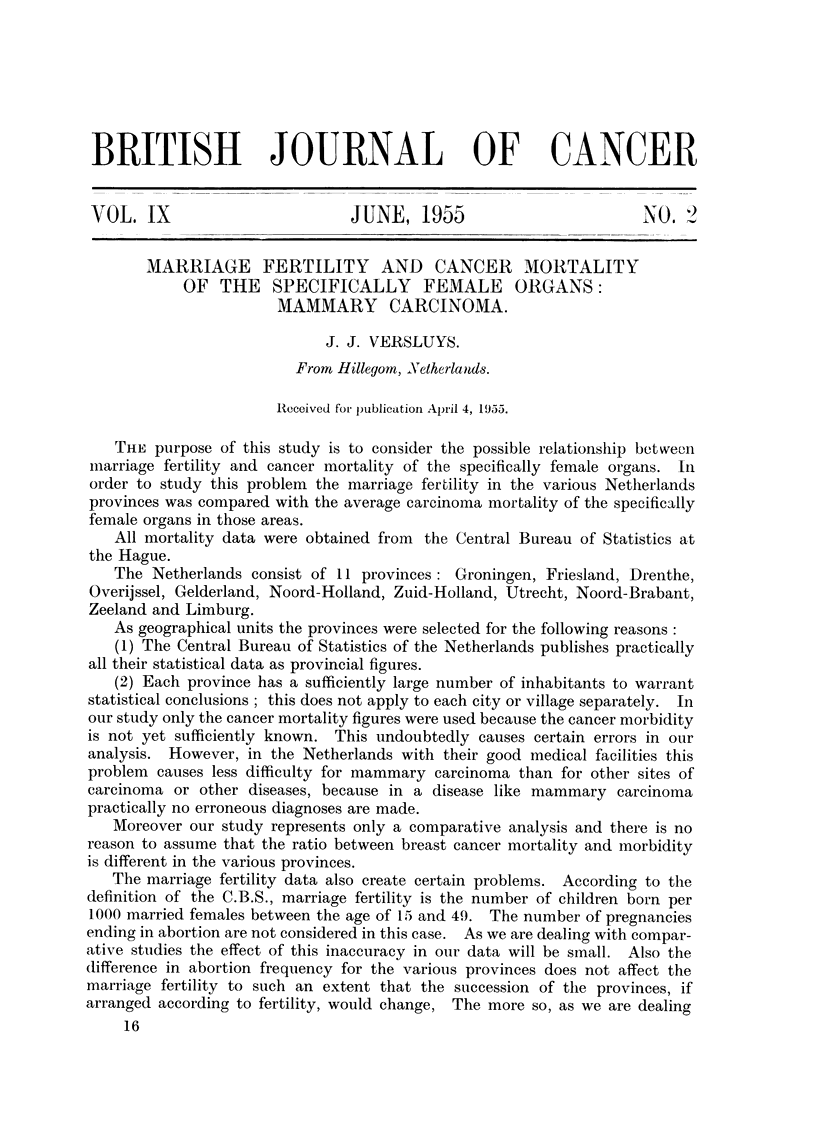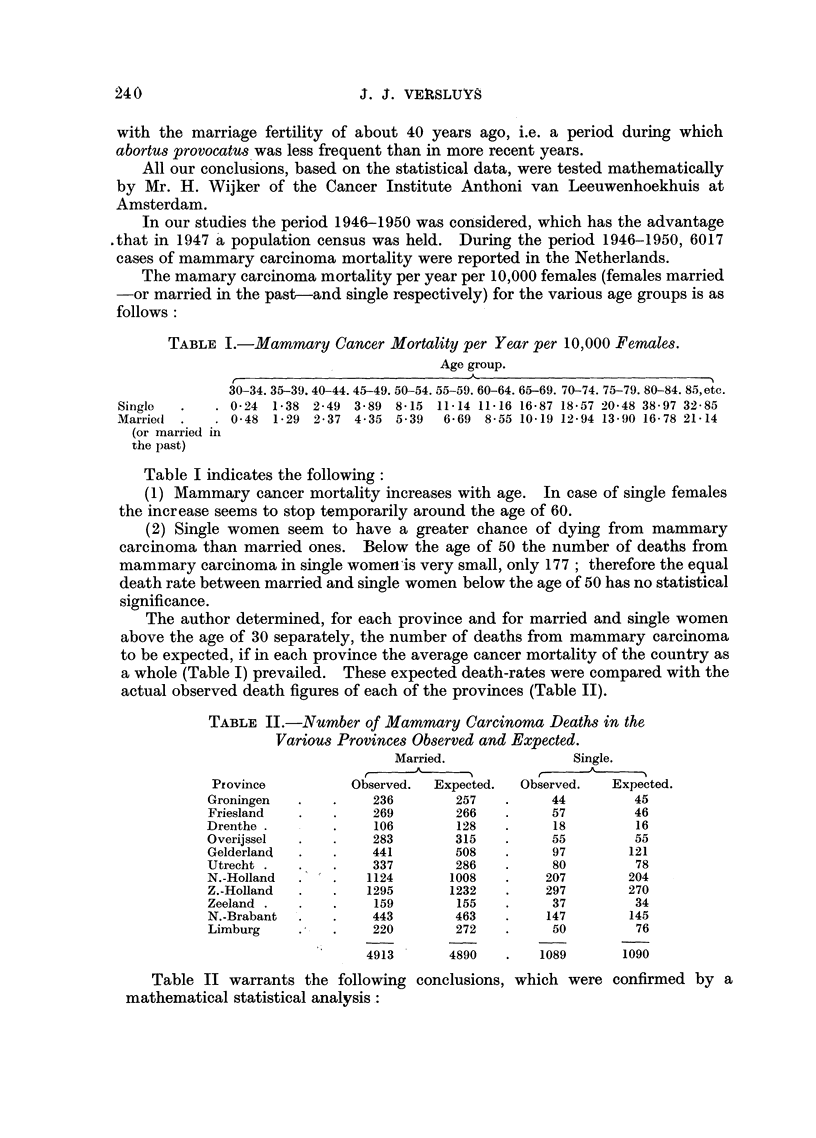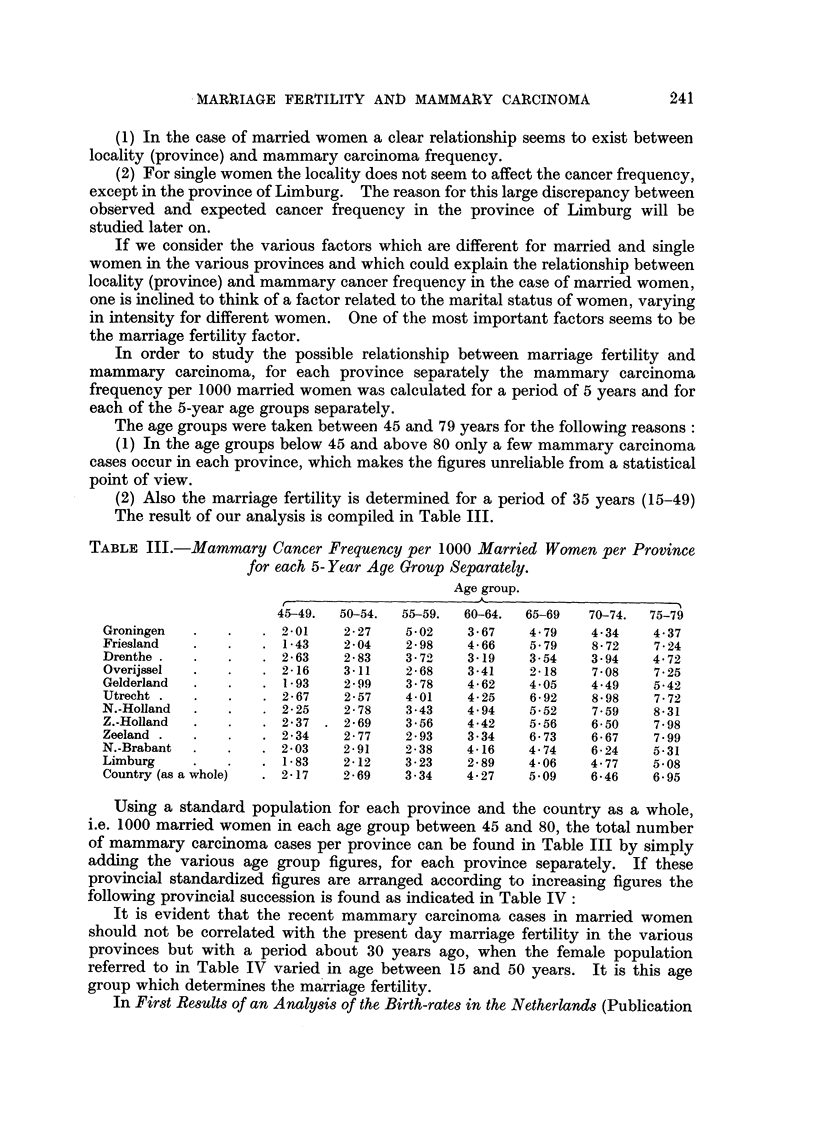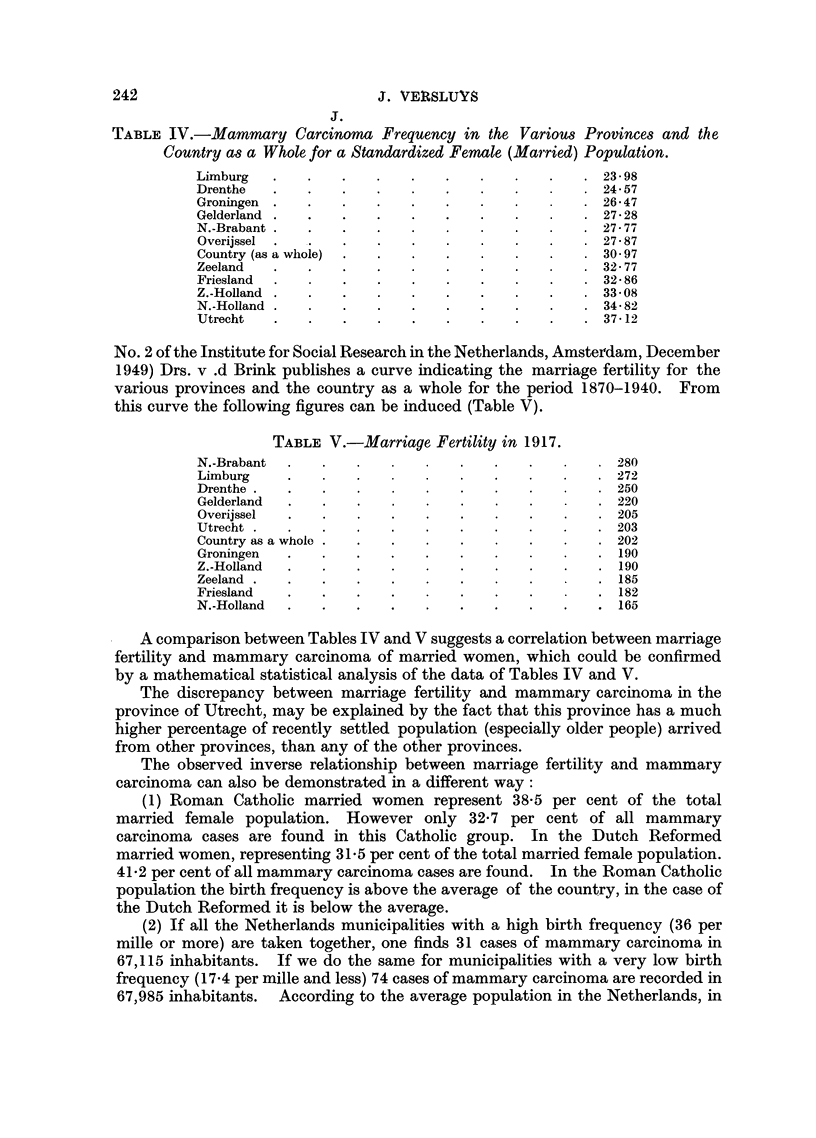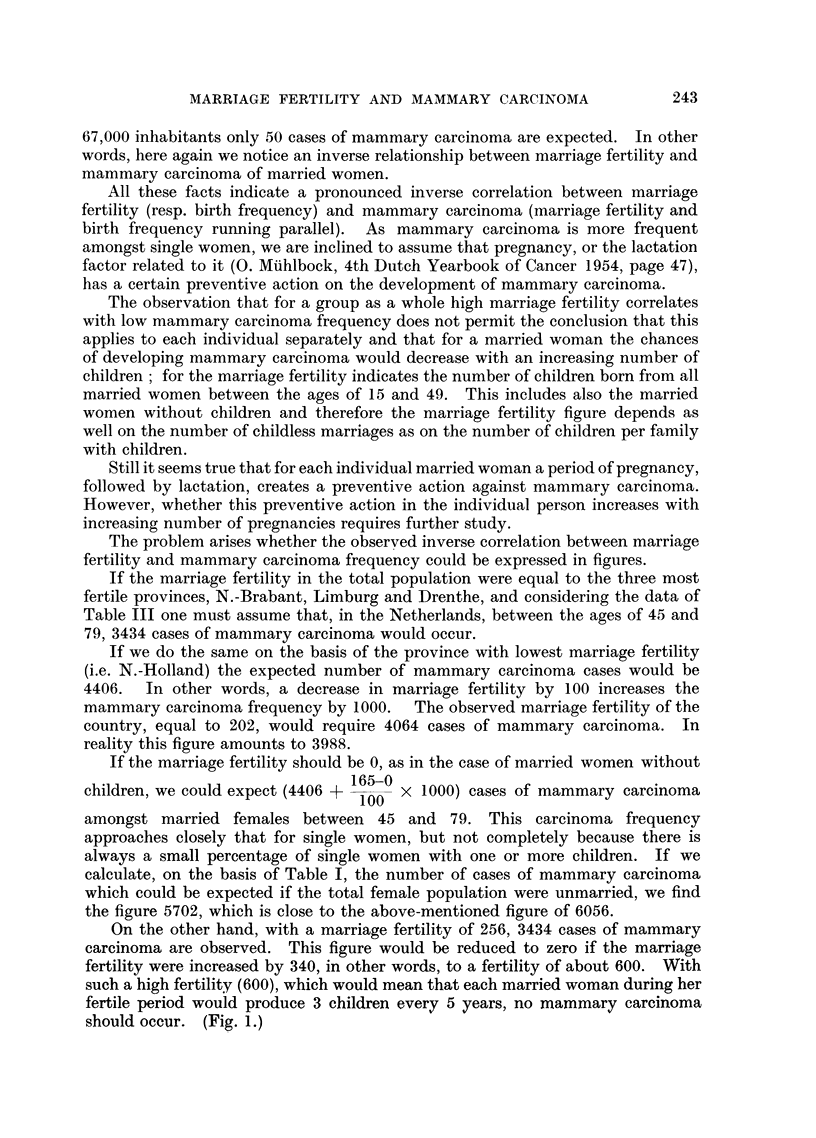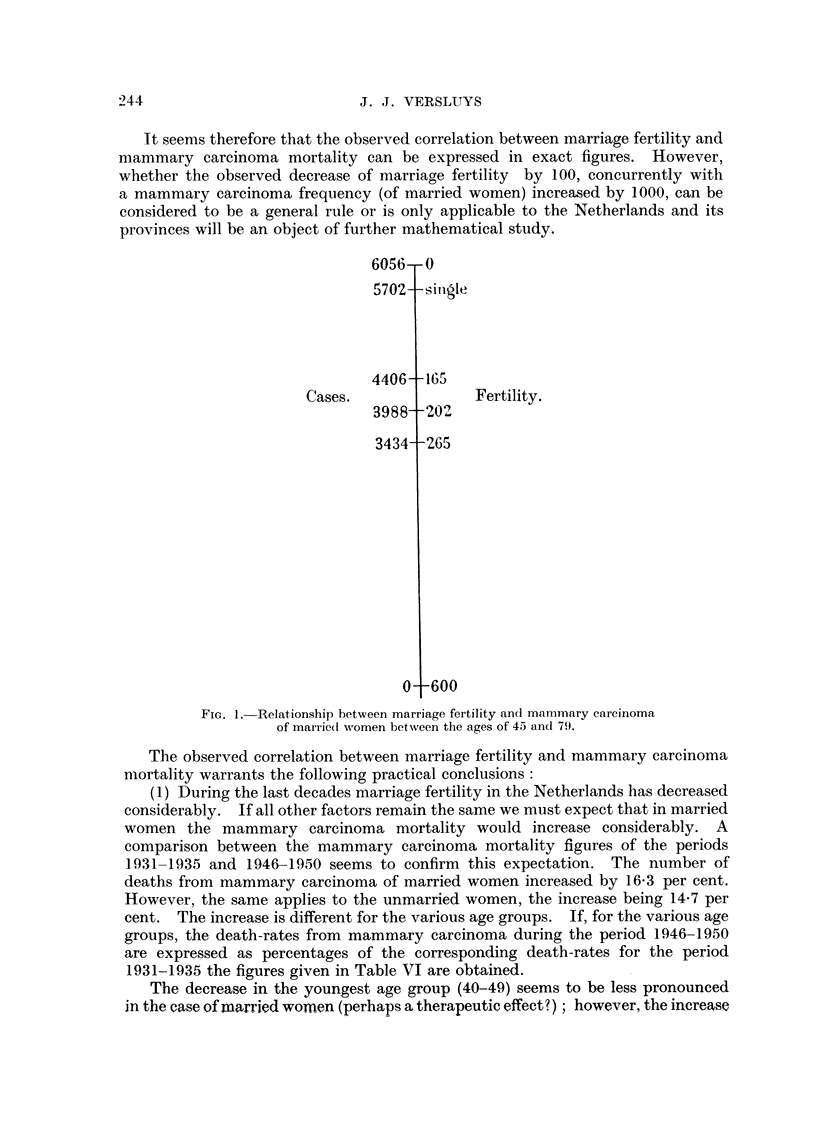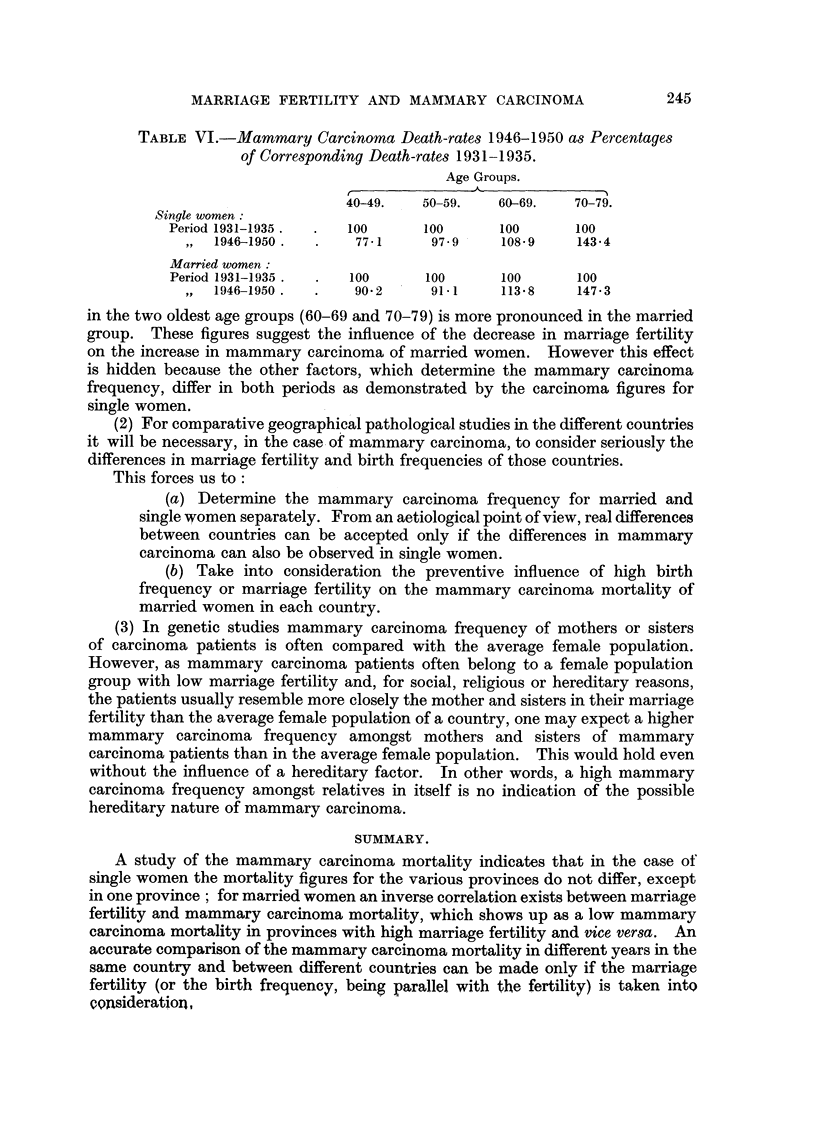# Marriage Fertility and Cancer Mortality of the Specifically Female Organs: Mammary Carcinoma

**DOI:** 10.1038/bjc.1955.20

**Published:** 1955-06

**Authors:** J. J. Versluys


BRITISH JOURNAL OF CANCER

VOL. IX JUNE, 1955 NO. 2

### MARRIAGE FERTILITY AND CANCER MORTALITY OF THE SPECIFICALLY FEMALE ORGANS: MAMMARY CARCINOMA.

J. J. VERSLUYS.

*From Hillegom, Netherlands.*

Received for publication April 4, 1955.

THE purpose of this study is to consider the possible relationship between marriage fertility and cancer mortality of the specifically female organs. In order to study this problem the marriage fertility in the various Netherlands provinces was compared with the average carcinoma mortality of the specifically female organs in those areas.

All mortality data were obtained from the Central Bureau of Statistics at the Hague.

The Netherlands consist of 11 provinces: Groningen, Friesland, Drenthe, Overijssel, Gelderland, Noord-Holland, Zuid-Holland, Utrecht, Noord-Brabant, Zeeland and Limburg.

As geographical units the provinces were selected for the following reasons:

(1) The Central Bureau of Statistics of the Netherlands publishes practically all their statistical data as provincial figures.

(2) Each province has a sufficiently large number of inhabitants to warrant statistical conclusions; this does not apply to each city or village separately. In our study only the cancer mortality figures were used because the cancer morbidity is not yet sufficiently known. This undoubtedly causes certain errors in our analysis. However, in the Netherlands with their good medical facilities this problem causes less difficulty for mammary carcinoma than for other sites of carcinoma or other diseases, because in a disease like mammary carcinoma practically no erroneous diagnoses are made.

Moreover our study represents only a comparative analysis and there is no reason to assume that the ratio between breast cancer mortality and morbidity is different in the various provinces.

The marriage fertility data also create certain problems. According to the definition of the C.B.S., marriage fertility is the number of children born per 1000 married females between the age of 15 and 49. The number of pregnancies ending in abortion are not considered in this case. As we are dealing with comparative studies the effect of this inaccuracy in our data will be small. Also the difference in abortion frequency for the various provinces does not affect the marriage fertility to such an extent that the succession of the provinces, if arranged according to fertility, would change, The more so, as we are dealing with the marriage fertility of about 40 years ago, i.e. a period during which *abortus provocatus* was less frequent than in more recent years.

All our conclusions, based on the statistical data, were tested mathematically by Mr. H. Wijker of the Cancer Institute Anthoni van Leeuwenhoekhuis at Amsterdam.

In our studies the period 1946–1950 was considered, which has the advantage that in 1947 a population census was held. During the period 1946–1950, 6017 cases of mammary carcinoma mortality were reported in the Netherlands.

The mamary carcinoma mortality per year per 10,000 females (females married —or married in the past—and single respectively) for the various age groups is as follows :

TABLE I.—*Mammary Cancer Mortality per Year per* 10,000 *Females.*

| | Age group. | | | | | | | | | | | |
|---|---|---|---|---|---|---|---|---|---|---|---|---|
| | 30–34. | 35–39. | 40–44. | 45–49. | 50–54. | 55–59. | 60–64. | 65–69. | 70–74. | 75–79. | 80–84. | 85, etc. |
| Single . . | 0·24 | 1·38 | 2·49 | 3·89 | 8·15 | 11·14 | 11·16 | 16·87 | 18·57 | 20·48 | 38·97 | 32·85 |
| Married . . (or married in the past) | 0·48 | 1·29 | 2·37 | 4·35 | 5·39 | 6·69 | 8·55 | 10·19 | 12·94 | 13·90 | 16·78 | 21·14 |

Table I indicates the following :

(1) Mammary cancer mortality increases with age. In case of single females the increase seems to stop temporarily around the age of 60.

(2) Single women seem to have a greater chance of dying from mammary carcinoma than married ones. Below the age of 50 the number of deaths from mammary carcinoma in single women is very small, only 177 ; therefore the equal death rate between married and single women below the age of 50 has no statistical significance.

The author determined, for each province and for married and single women above the age of 30 separately, the number of deaths from mammary carcinoma to be expected, if in each province the average cancer mortality of the country as a whole (Table I) prevailed. These expected death-rates were compared with the actual observed death figures of each of the provinces (Table II).

TABLE II.—*Number of Mammary Carcinoma Deaths in the Various Provinces Observed and Expected.*

| | Married. | | Single. | |
|---|---|---|---|---|
| Province | Observed. | Expected. | Observed. | Expected. |
| Groningen . . | 236 | 257 | 44 | 45 |
| Friesland . . | 269 | 266 | 57 | 46 |
| Drenthe . . | 106 | 128 | 18 | 16 |
| Overijssel . . | 283 | 315 | 55 | 55 |
| Gelderland . . | 441 | 508 | 97 | 121 |
| Utrecht . . | 337 | 286 | 80 | 78 |
| N.-Holland . . | 1124 | 1008 | 207 | 204 |
| Z.-Holland . . | 1295 | 1232 | 297 | 270 |
| Zeeland . . | 159 | 155 | 37 | 34 |
| N.-Brabant . . | 443 | 463 | 147 | 145 |
| Limburg . . | 220 | 272 | 50 | 76 |
| | 4913 | 4890 | 1089 | 1090 |

Table II warrants the following conclusions, which were confirmed by a mathematical statistical analysis :

(1) In the case of married women a clear relationship seems to exist between locality (province) and mammary carcinoma frequency.

(2) For single women the locality does not seem to affect the cancer frequency, except in the province of Limburg. The reason for this large discrepancy between observed and expected cancer frequency in the province of Limburg will be studied later on.

If we consider the various factors which are different for married and single women in the various provinces and which could explain the relationship between locality (province) and mammary cancer frequency in the case of married women, one is inclined to think of a factor related to the marital status of women, varying in intensity for different women. One of the most important factors seems to be the marriage fertility factor.

In order to study the possible relationship between marriage fertility and mammary carcinoma, for each province separately the mammary carcinoma frequency per 1000 married women was calculated for a period of 5 years and for each of the 5-year age groups separately.

The age groups were taken between 45 and 79 years for the following reasons :

(1) In the age groups below 45 and above 80 only a few mammary carcinoma cases occur in each province, which makes the figures unreliable from a statistical point of view.

(2) Also the marriage fertility is determined for a period of 35 years (15–49)

The result of our analysis is compiled in Table III.

TABLE III.—*Mammary Cancer Frequency per* 1000 *Married Women per Province for each* 5-*Year Age Group Separately.*

| | Age group. | | | | | | |
|---|---|---|---|---|---|---|---|
| | 45–49. | 50–54. | 55–59. | 60–64. | 65–69 | 70–74. | 75–79 |
| Groningen | 2·01 | 2·27 | 5·02 | 3·67 | 4·79 | 4·34 | 4·37 |
| Friesland | 1·43 | 2·04 | 2·98 | 4·66 | 5·79 | 8·72 | 7·24 |
| Drenthe | 2·63 | 2·83 | 3·72 | 3·19 | 3·54 | 3·94 | 4·72 |
| Overijssel | 2·16 | 3·11 | 2·68 | 3·41 | 2·18 | 7·08 | 7·25 |
| Gelderland | 1·93 | 2·99 | 3·78 | 4·62 | 4·05 | 4·49 | 5·42 |
| Utrecht | 2·67 | 2·57 | 4·01 | 4·25 | 6·92 | 8·98 | 7·72 |
| N.-Holland | 2·25 | 2·78 | 3·43 | 4·94 | 5·52 | 7·59 | 8·31 |
| Z.-Holland | 2·37 | 2·69 | 3·56 | 4·42 | 5·56 | 6·50 | 7·98 |
| Zeeland | 2·34 | 2·77 | 2·93 | 3·34 | 6·73 | 6·67 | 7·99 |
| N.-Brabant | 2·03 | 2·91 | 2·38 | 4·16 | 4·74 | 6·24 | 5·31 |
| Limburg | 1·83 | 2·12 | 3·23 | 2·89 | 4·06 | 4·77 | 5·08 |
| Country (as a whole) | 2·17 | 2·69 | 3·34 | 4·27 | 5·09 | 6·46 | 6·95 |

Using a standard population for each province and the country as a whole, i.e. 1000 married women in each age group between 45 and 80, the total number of mammary carcinoma cases per province can be found in Table III by simply adding the various age group figures, for each province separately. If these provincial standardized figures are arranged according to increasing figures the following provincial succession is found as indicated in Table IV :

It is evident that the recent mammary carcinoma cases in married women should not be correlated with the present day marriage fertility in the various provinces but with a period about 30 years ago, when the female population referred to in Table IV varied in age between 15 and 50 years. It is this age group which determines the marriage fertility.

In *First Results of an Analysis of the Birth-rates in the Netherlands* (Publication

J.

TABLE IV.—*Mammary Carcinoma Frequency in the Various Provinces and the Country as a Whole for a Standardized Female (Married) Population.*

| | |
|---|---|
| Limburg | 23·98 |
| Drenthe | 24·57 |
| Groningen | 26·47 |
| Gelderland | 27·28 |
| N.-Brabant | 27·77 |
| Overijssel | 27·87 |
| Country (as a whole) | 30·97 |
| Zeeland | 32·77 |
| Friesland | 32·86 |
| Z.-Holland | 33·08 |
| N.-Holland | 34·82 |
| Utrecht | 37·12 |

No. 2 of the Institute for Social Research in the Netherlands, Amsterdam, December 1949) Drs. v .d Brink publishes a curve indicating the marriage fertility for the various provinces and the country as a whole for the period 1870–1940. From this curve the following figures can be induced (Table V).

TABLE V.—*Marriage Fertility in* 1917.

| | |
|---|---|
| N.-Brabant | 280 |
| Limburg | 272 |
| Drenthe | 250 |
| Gelderland | 220 |
| Overijssel | 205 |
| Utrecht | 203 |
| Country as a whole | 202 |
| Groningen | 190 |
| Z.-Holland | 190 |
| Zeeland | 185 |
| Friesland | 182 |
| N.-Holland | 165 |

A comparison between Tables IV and V suggests a correlation between marriage fertility and mammary carcinoma of married women, which could be confirmed by a mathematical statistical analysis of the data of Tables IV and V.

The discrepancy between marriage fertility and mammary carcinoma in the province of Utrecht, may be explained by the fact that this province has a much higher percentage of recently settled population (especially older people) arrived from other provinces, than any of the other provinces.

The observed inverse relationship between marriage fertility and mammary carcinoma can also be demonstrated in a different way :

(1) Roman Catholic married women represent 38·5 per cent of the total married female population. However only 32·7 per cent of all mammary carcinoma cases are found in this Catholic group. In the Dutch Reformed married women, representing 31·5 per cent of the total married female population. 41·2 per cent of all mammary carcinoma cases are found. In the Roman Catholic population the birth frequency is above the average of the country, in the case of the Dutch Reformed it is below the average.

(2) If all the Netherlands municipalities with a high birth frequency (36 per mille or more) are taken together, one finds 31 cases of mammary carcinoma in 67,115 inhabitants. If we do the same for municipalities with a very low birth frequency (17·4 per mille and less) 74 cases of mammary carcinoma are recorded in 67,985 inhabitants. According to the average population in the Netherlands, in

67,000 inhabitants only 50 cases of mammary carcinoma are expected. In other words, here again we notice an inverse relationship between marriage fertility and mammary carcinoma of married women.

All these facts indicate a pronounced inverse correlation between marriage fertility (resp. birth frequency) and mammary carcinoma (marriage fertility and birth frequency running parallel). As mammary carcinoma is more frequent amongst single women, we are inclined to assume that pregnancy, or the lactation factor related to it (O. Mühlbock, 4th Dutch Yearbook of Cancer 1954, page 47), has a certain preventive action on the development of mammary carcinoma.

The observation that for a group as a whole high marriage fertility correlates with low mammary carcinoma frequency does not permit the conclusion that this applies to each individual separately and that for a married woman the chances of developing mammary carcinoma would decrease with an increasing number of children ; for the marriage fertility indicates the number of children born from all married women between the ages of 15 and 49. This includes also the married women without children and therefore the marriage fertility figure depends as well on the number of childless marriages as on the number of children per family with children.

Still it seems true that for each individual married woman a period of pregnancy, followed by lactation, creates a preventive action against mammary carcinoma. However, whether this preventive action in the individual person increases with increasing number of pregnancies requires further study.

The problem arises whether the observed inverse correlation between marriage fertility and mammary carcinoma frequency could be expressed in figures.

If the marriage fertility in the total population were equal to the three most fertile provinces, N.-Brabant, Limburg and Drenthe, and considering the data of Table III one must assume that, in the Netherlands, between the ages of 45 and 79, 3434 cases of mammary carcinoma would occur.

If we do the same on the basis of the province with lowest marriage fertility (i.e. N.-Holland) the expected number of mammary carcinoma cases would be 4406. In other words, a decrease in marriage fertility by 100 increases the mammary carcinoma frequency by 1000. The observed marriage fertility of the country, equal to 202, would require 4064 cases of mammary carcinoma. In reality this figure amounts to 3988.

If the marriage fertility should be 0, as in the case of married women without children, we could expect $(4406 + \frac{165-0}{100} \times 1000)$ cases of mammary carcinoma amongst married females between 45 and 79. This carcinoma frequency approaches closely that for single women, but not completely because there is always a small percentage of single women with one or more children. If we calculate, on the basis of Table I, the number of cases of mammary carcinoma which could be expected if the total female population were unmarried, we find the figure 5702, which is close to the above-mentioned figure of 6056.

On the other hand, with a marriage fertility of 256, 3434 cases of mammary carcinoma are observed. This figure would be reduced to zero if the marriage fertility were increased by 340, in other words, to a fertility of about 600. With such a high fertility (600), which would mean that each married woman during her fertile period would produce 3 children every 5 years, no mammary carcinoma should occur. (Fig. 1.)

It seems therefore that the observed correlation between marriage fertility and mammary carcinoma mortality can be expressed in exact figures. However, whether the observed decrease of marriage fertility by 100, concurrently with a mammary carcinoma frequency (of married women) increased by 1000, can be considered to be a general rule or is only applicable to the Netherlands and its provinces will be an object of further mathematical study.

FIG. 1.—Relationship between marriage fertility and mammary carcinoma of married women between the ages of 45 and 79.

The observed correlation between marriage fertility and mammary carcinoma mortality warrants the following practical conclusions :

(1) During the last decades marriage fertility in the Netherlands has decreased considerably. If all other factors remain the same we must expect that in married women the mammary carcinoma mortality would increase considerably. A comparison between the mammary carcinoma mortality figures of the periods 1931–1935 and 1946–1950 seems to confirm this expectation. The number of deaths from mammary carcinoma of married women increased by 16·3 per cent. However, the same applies to the unmarried women, the increase being 14·7 per cent. The increase is different for the various age groups. If, for the various age groups, the death-rates from mammary carcinoma during the period 1946–1950 are expressed as percentages of the corresponding death-rates for the period 1931–1935 the figures given in Table VI are obtained.

The decrease in the youngest age group (40–49) seems to be less pronounced in the case of married women (perhaps a therapeutic effect?) ; however, the increase

TABLE VI.—*Mammary Carcinoma Death-rates* 1946–1950 *as Percentages of Corresponding Death-rates* 1931–1935.

| | Age Groups. | | | |
|---|---|---|---|---|
| | 40–49. | 50–59. | 60–69. | 70–79. |
| *Single women :* | | | | |
| Period 1931–1935 . . | 100 | 100 | 100 | 100 |
| „ 1946–1950 . . | 77·1 | 97·9 | 108·9 | 143·4 |
| *Married women :* | | | | |
| Period 1931–1935 . . | 100 | 100 | 100 | 100 |
| „ 1946–1950 . . | 90·2 | 91·1 | 113·8 | 147·3 |

in the two oldest age groups (60–69 and 70–79) is more pronounced in the married group. These figures suggest the influence of the decrease in marriage fertility on the increase in mammary carcinoma of married women. However this effect is hidden because the other factors, which determine the mammary carcinoma frequency, differ in both periods as demonstrated by the carcinoma figures for single women.

(2) For comparative geographical pathological studies in the different countries it will be necessary, in the case of mammary carcinoma, to consider seriously the differences in marriage fertility and birth frequencies of those countries.

This forces us to :

(*a*) Determine the mammary carcinoma frequency for married and single women separately. From an aetiological point of view, real differences between countries can be accepted only if the differences in mammary carcinoma can also be observed in single women.

(*b*) Take into consideration the preventive influence of high birth frequency or marriage fertility on the mammary carcinoma mortality of married women in each country.

(3) In genetic studies mammary carcinoma frequency of mothers or sisters of carcinoma patients is often compared with the average female population. However, as mammary carcinoma patients often belong to a female population group with low marriage fertility and, for social, religious or hereditary reasons, the patients usually resemble more closely the mother and sisters in their marriage fertility than the average female population of a country, one may expect a higher mammary carcinoma frequency amongst mothers and sisters of mammary carcinoma patients than in the average female population. This would hold even without the influence of a hereditary factor. In other words, a high mammary carcinoma frequency amongst relatives in itself is no indication of the possible hereditary nature of mammary carcinoma.

### SUMMARY.

A study of the mammary carcinoma mortality indicates that in the case of single women the mortality figures for the various provinces do not differ, except in one province ; for married women an inverse correlation exists between marriage fertility and mammary carcinoma mortality, which shows up as a low mammary carcinoma mortality in provinces with high marriage fertility and *vice versa*. An accurate comparison of the mammary carcinoma mortality in different years in the same country and between different countries can be made only if the marriage fertility (or the birth frequency, being parallel with the fertility) is taken into consideration.